# Dopaminergic antagonists inhibit bile chemotaxis of adult *Clonorchis sinensis* and its egg production

**DOI:** 10.1371/journal.pntd.0008220

**Published:** 2020-03-30

**Authors:** Fuhong Dai, Jin-Ho Song, Yeon Pyo Hong, Xuelian Bai, Woon-Mok Sohn, Sung-Jong Hong

**Affiliations:** 1 Department of Medical Environmental Biology, Chung-Ang University College of Medicine, Seoul, Republic of Korea; 2 Department of Parasitology, School of Biology and Basic Medical Sciences, Medical College, Soochow University, Suzhou, Jiangsu, PR China; 3 Department of Pharmacology, Chung-Ang University College of Medicine, Seoul, Republic of Korea; 4 Department of Preventive Medicine, Chung-Ang University College of Medicine, Seoul, Republic of Korea; 5 Clinical Medicine Laboratory, Affiliated Hospital of Binzhou Medical University, Binzhou, Shandong, PR China; 6 Department of Parasitology and Institute of Health Sciences, Gyeongsang National University College of Medicine, Jinju, Republic of Korea; Seoul National University College of Medicine, REPUBLIC OF KOREA

## Abstract

Human clonorchiasis, caused by *Clonorchis sinensis*, is endemic in East Asian countries. *C*. *sinensis* metacercariae excyst in the duodenum of mammalian hosts, migrate to the intrahepatic bile duct, and mature into adults in the milieu of bile. We have previously shown that newly excysted juvenile *C*. *sinensis* move chemotactically toward bile and bile acids. Here, the chemotactic behavior of adult *C*. *sinensis* (CsAd) toward bile and bile acids was investigated. CsAds moved toward 0.05–5% bile and were most attracted to 0.5% bile but moved away from 10% bile. Upon exposure to 1–10% bile, CsAds eventually stopped moving and then died quickly. Among bile acids, CsAds showed strong chemotaxis toward cholic acid (CA) and deoxycholic acid. On the contrary, CsAds repelled from lithocholic acid (LCA). Moreover, at higher than 10 mM LCA, CsAds became sluggish and eventually died. Dopamine D_1_ receptor antagonists (LE-300 and SKF-83566), D_2/3_ receptor antagonists (raclopride and its derivative CS-49612), and a dopamine re-uptake inhibitor inhibited CA-induced chemotaxis of CsAds almost completely. Clinically used antipsychotic drugs, namely chlorpromazine, haloperidol, and clozapine, are dopaminergic antagonists and are secreted into bile. They completely inhibited chemotaxis of CsAds toward CA. At the maximum doses used to treat patients, the three tested medicines only expelled 2–12% of CsAds from the experimentally infected rabbits, but reduced egg production by 64–79%. Thus, antipsychotic medicines with dopaminergic antagonism could be considered as new anthelmintic candidates for human *C*. *sinensis* infections.

## Author summary

The liver fluke, *Clonorchis sinensis*, is endemic in East Asian countries. People get infected through consumption of raw freshwater fish carrying *C*. *sinensis* metacercaria, an infective form to human. The metacercaria excysts in the human duodenum and the juvenile fluke migrates into the intrahepatic bile duct following chemical cues provided by bile. The juvenile fluke matures in the intrahepatic bile duct, and the adult fluke causes inflammatory injuries to the bile duct, which can eventually lead to bile duct cancer. Bile plays a critical role in the fluke’s life in the human host. In this study, we demonstrated that the adult *C*. *sinensis* fluke was attracted by bile and bile acids, such as cholic acid and deoxycholic acid, but repelled by lithocholic acid. The chemotactic attraction of the fluke to cholic acid was suppressed by experimental dopaminergic antagonists. Clinically used antipsychotic drugs that are dopaminergic antagonists, namely chlorpromazine, haloperidol, and clozapine, also strongly suppressed the chemotaxis. While the drugs elicited a minor response of fluke expulsion from the bile ducts of infected rabbits, they significantly reduced egg production. The results could be utilized to develop effective drugs to treat *C*. *sinensis* infection.

## Introduction

*Clonorchis sinensis*, Chinese liver fluke, causes clonorchiasis endemic in Asian countries and infected approximately 35 million people [1, 2]. Humans are infected when they consume raw freshwater fish contaminated with *C*. *sinensis* metacercariae. The ingested metacercariae excyst in the duodenum and pass through the ampulla of Vater and common bile duct to enter the intrahepatic bile ducts. Once living inside the intrahepatic bile ducts, *C*. *sinensis* adults give rise to jaundice, and provide continuous physical and chemical stimuli. The metabolic and secretory products damage the biliary epithelium and cause fibrosis of the bile duct wall. Chronic clonorchiasis could, in some cases, cause complications, such as cholecystitis, cholelithiasis, biliary cirrhosis, pyogenic cholangitis, and even cholangiocarcinoma [2–5].

Chemotactic behavior for survival and navigation to a specific host is not uncommon in helminthic trematodes and nematodes. *Schistosoma mansoni*, a blood fluke, is chemotactically attracted to d-glucose and l-arginine [6]. *Diplostomum spathaceum* cercariae recognize fish by sensing monosaccharides on the host surface, and their penetration into the host skin is stimulated by hydrophilic macromolecules and lipids [7]. *Caenorhabditis elegans* is attracted by either sodium acetate or diacetyl [8].

Adult *C*. *sinensis* parasitize in the bile ducts of mammals, being continuously exposed to bile throughout their lives. *C*. *sinensis* is believed to have evolved bile-tolerance, to withstand living in such an extreme environment. When *C*. *sinensis* newly-excysted juveniles (CsNEJs) are exposed to bile, the expression of genes related to energy production, cell apoptosis, and proliferation increase [9]. Furthermore, CsNEJs are more active and survive longer in media containing bile than that without [10].

Bile is a strong chemotactic factor and attracts CsNEJs to move toward the bile ducts. When bile is secreted in rabbits, CsNEJs migrate up from the duodenum to the intrahepatic bile ducts as early as 7–9 min after secretion [11]. Of the bile components, cholic acid (CA) acts as a strong attractant to CsNEJs, and lithocholic acid (LCA) acts as a repellent [12]. CA-induced chemotaxis of CsNEJs is potently inhibited by dopaminergic antagonists, suggesting dopaminergic neurons transduce and control chemotactic behavior [13].

While the chemotaxis of CsNEJs to bile and bile acids and its neuronal signaling have been elucidated, the influence of bile on *C*. *sinensis* adults (CsAds) remains unknown. As CsAds live in a bile-rich environment, it is assumed they continuously interact with bile components. In the present study, we investigated the chemotactic behavior of CsAds to bile and bile acids and its inhibition by dopaminergic antagonists. Clinical antipsychotic drugs with dopamine antagonism were also investigated for their potential to expel CsAds from infected rabbits.

## Materials and methods

### Ethics statement

Rabbits (female New Zealand White, 2.2–2.4 kg) were purchased from Samtako Bio Korea Inc. (Osan, Korea). The animal care and use protocol was reviewed and approved by the Institutional Animal Care and Use Committee (IACUC) at Chung-Ang University (Approval No. CAU-2014-0024), in accordance with Association for Assessment and Accreditation of Laboratory Animal Care (AAALAC) International Animal Care policies. Rabbits were cared and handled in the Chung-Ang University Animal Facility in accordance with the National Animal Care Policies (Accredited Unit, Korea FDA; Unit number 36).

### Preparation of *C*. *sinensis* metacercariae and adults

Freshwater fishes *Pseudorasbora parva* and *Pungtungia herzi*, the second intermediate hosts of *C*. *sinensis*, were collected from the Nam River, Jinju, Gyeongsangnam-do, Korea. Fish were ground and digested in artificial gastric juice (0.4% pepsin, pH 1.0, MP Biochemicals Co., Solon, OH) for 2 h at 37°C. The coarse matter was removed from the digested content through a 1 mm mesh-diameter sieve. The filtrate was allowed to settle and was washed several times by discarding the upper half and adding 0.85% saline. The *C*. *sinensis* metacercariae were collected under a dissecting microscope and stored at 4°C in phosphate-buffered saline (PBS) containing antibiotics and antimycotics (GIBCO, Invitrogen, Carlsbad, CA) until use [14].

Three hundred *C*. *sinensis* metacercariae were orally administered to each rabbit, twice within a one-week interval. Two months later, the rabbits were sacrificed and *C*. *sinensis* adult flukes were recovered from the bile ducts.

### Survival and activity of CsAds *in vitro*

The CsAds were incubated, 5 flukes each, in a petri-dish (35 × 10 mm, Falcon, Corning, NY) containing 5 mL of 1× Locke’s solution. The survival rate and activity of the adult flukes were checked under a stereomicroscope every 1 h for 12 h, and then at 18, 24, 30, 42, 54, 66, 78, 90, 102, and 126 h (S1 Fig). The solution was replaced with fresh solution at every time point. A worm was judged as dead if it did not show any reaction when stirred gently with the soft end of a wooden applicator. The activity of a fluke was scored arbitrarily between 0–5; 5 at the beginning and 0 when dead. This assay was carried out in duplicate at 37°C and 60% humidity in a walk-in incubator (Jisico, Seoul, Korea).

### Regurgitating bile from the intestinal ceca

The CsAds were incubated, 5 flukes each, in a petri dish containing 5 mL of 1× Locke’s solution. The incubation solution was collected and replaced every 1 h for 12 h, and then at 18, 24, 30, 42, 54, and 66 h. The solutions were assayed for bile concentration using a Bile Acids Enzymatic Colorimetric Manual Kit according to the manufacturer’s instruction (Randox, Crumlin, UK). The assay principle is, in brief, that 3α-hydroxy bile acid reacts with α-hydroxysteroid dehydrogenase and nitrotetrazolium blue, forming formazan, which has an absorbance wavelength maximum at 546 nm; the absorbance is directly proportional to the bile acid concentration (S2 Fig).

### Chemotactic migration of CsAds

**Fig 1:**
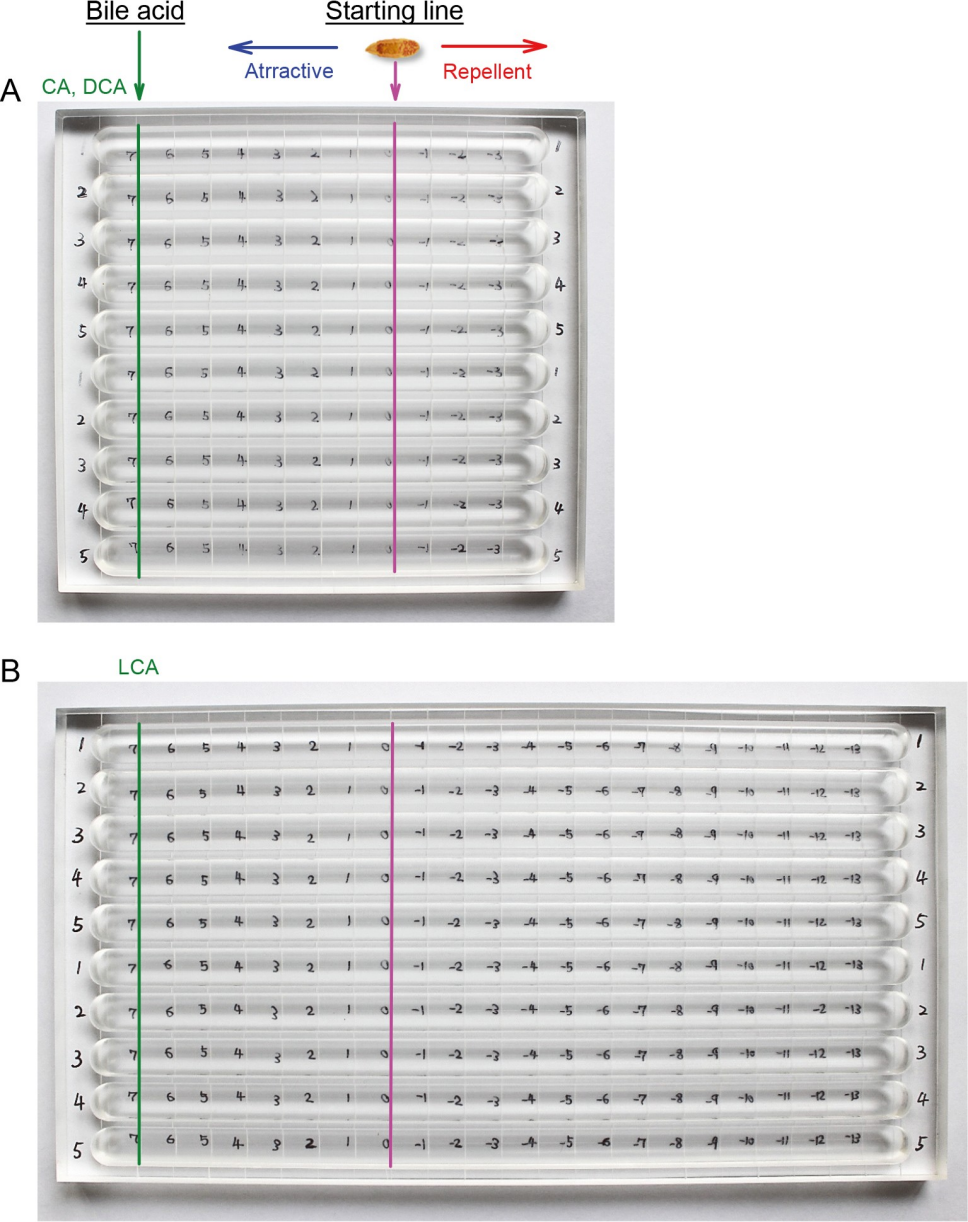
Two types of chemotaxis assay panels. (A) A short assay panel for chemo-attractants, cholic acid (CA) and deoxycholic acid (DCA). (B) A long assay panel for the chemo-repellent lithocholic acid (LCA). An adult *C*. *sinensis* fluke was placed at the starting line and bile acid was dropped at the left end of a trough. The numbers inside the trough are in cm.

The CsAds, having regurgitated all contaminating-host bile as described above, were used to evaluate chemotaxis. The chemotaxis assay panel was custom-made with slight modification to the one used for CsNEJs [12]. Ten half-round troughs that were 12 or 22 cm long, 1 cm wide, and 0.8 cm deep, were carved into a polycarbonate block (Fig 1). Each trough was graduated in a mm-scale on the bottom surface. Two sets of assays with graded bile concentrations were carried out at a time. Each set had 5 troughs with 1 CsAd in each trough. The CsAd was placed with the oral sucker toward the bile/bile acid, and the ventral sucker downward, simulating the movement of flukes by making the two suckers function in tandem, as is seen in an inchworm.

For the bile chemotactic assay, a 12-cm-long assay panel was employed, and each trough was filled with 6 mL of 1× Locke’s solution (Fig 1A). The CsAd was placed at the starting line of the trough. Forty-microliters of 0.05–10% bovine bile (Sigma-Aldrich, St. Louis, MO) was placed 7 cm left of the starting line. Migration distance, body shape, and behavior of the CsAds were observed and recorded. For CA or deoxycholic acid (DCA), 4 μL of 25–200 mM solution was used. For LCA, a 22-cm-long chemotactic assay trough was used (Fig 1B). The trough was filled with 12 mL of 1× Locke’s solution and 4 μL of 2.5–20 mM LCA (Sigma-Aldrich) was dropped 7 cm from the starting line.

### Effect of dopaminergic antagonists to CsAds

To assess the effects of dopaminergic inhibitors on the chemotaxis of CsAds toward CA, a trough was filled with 6 mL 1× Locke’s solution containing a test inhibitor. One CsAd was placed at the starting line, incubated for 10 min, and then stimulated with 4 μL of 100 mM CA dropped 7 cm from the starting line. To assess the effects of clinical antipsychotic drugs, a test drug was mixed with CA to mimic a secreting condition in the bile duct.

All chemicals were purchased from Sigma-Aldrich Co. (St. Louis, MO) unless otherwise specified. Bile and bile acids were dissolved in dimethyl sulfoxide (DMSO). Test chemicals were dissolved in 99.5% ethanol at a stock concentration of 10 mM and diluted to test concentrations in 1× Locke’s solution. The solvents were used as negative controls.

### *In vivo* effects of antipsychotic drugs to CsAds

Each experimental group included five rabbits. Rabbits were infected orally with 400 *C*. *sinensis* metacercariae (200 metacercariae twice at a week interval). After 45 days, an antipsychotic drug, namely chlorpromazine, haloperidol, or clozapine, was orally administered. Drug-dosages were determined based on human dosages, and drugs were administered twice a day for two consecutive days (chlorpromazine 39 mg/day, haloperidol 1.5 mg/day, and clozapine 44 mg/day). A single dose of each drug was dissolved in 0.5 mL ethanol and diluted in 1 mL dH_2_O. Praziquantel, 150 mg/kg, was orally administered once.

Rabbits were sacrificed 5 days after the last drug administration, and CsAds were recovered from the bile ducts. The normal control rabbits were infected with *C*. *sinensis* but not treated with any drug. The positive control rabbits were infected with *C*. *sinensis* and treated with praziquantel.

Worm burden in clonorchiasis can be predicted from the number of eggs per gram of feces (EPG) [15]. The experimental rabbits’ feces were collected once a day for 3 days before the first drug administration, and for 3 days after the last drug administration. One gram of feces was crushed into small pieces in 10 mL of water in a tube, shaken vigorously or vortexed to disperse thoroughly, and allowed to settle overnight at 4°C. The tube was shaken vigorously again to disperse the *C*. *sinensis* eggs in the mixture. Debris was removed by filtering through two-layer gauze. The tube was rinsed with 5 mL of water and filtered into the previous tube. From the filtrate, 50 μL was taken and put onto a glass slide, and *C*. *sinensis* eggs were counted under a light microscope. Microscopy was performed in triplicate on each fecal sample. The egg counts were converted into EPG by multiplying by 300 (×300).

### Data analysis

The assays on bile chemotaxis were performed in triplicate, with different sets of 5 CsAds. Results are presented as mean ± standard error of the mean (SEM). Data were analyzed using the Student's *t*-test, and values of *p* < 0.05 are considered statistically significant.

## Results

### CsAds were active and survive long *in vitro*

The CsAds displayed a good survival rate for the first 66 h in 1× Locke’s solution, before decreasing after 78 h, until no viable flukes remained after 126 h (S1 Fig). The CsAds moved actively, extending and retracting the anterior part between the oral and ventral suckers for 42 h, whereafter activity decreased up to 78 h; after 78 h several flukes began to die, and live flukes responded dully to stirring stimuli. Based on these survival results, all bile chemotactic assays were carried out on active CsAds in 1× Locke’s solution for 3 h.

### CsAds regurgitated and emptied intestinal bile in short time

Due to inhabiting the host’s intrahepatic bile duct, CsAd intestines are full of the host’s bile. When the CsAds regurgitate the intestinal bile, the incubation solution becomes contaminated and interferes with the chemotactic response of CsAds to exogenously applied bile. Thus, CsAd intestines should be emptied of bile before chemotactic assays. The CsAds regurgitated a large amount of bile for 6–10 h during incubation (S2 Fig). The amount of regurgitated bile decreased by 24 h and remained low until 66 h. Based on this finding, the CsAds were pre-incubated for 24 h before being prepared for bile chemotaxis assays.

### CsAds chemotactically migrated to bile and bile acids

**Fig 2:**
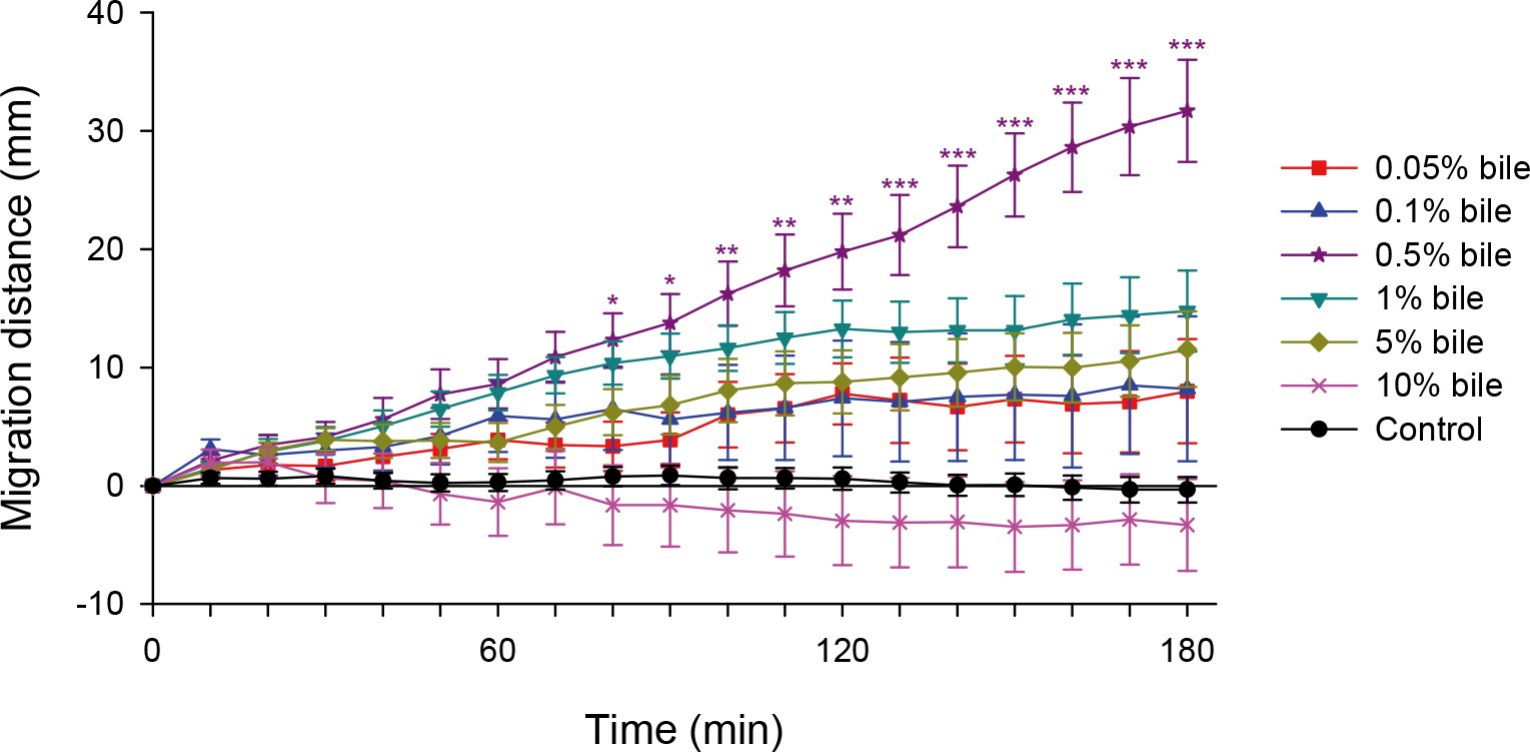
Chemotaxis of *C*. *sinensis* adult flukes to bile. Chemotactic migration distance of *C*. *sinensis* adults (CsAds) in response to various concentrations of bile was measured every 10 min for 3 h. Asterisks indicate * *p* < 0.05, ** *p* < 0.01, and *** *p* < 0.001 compared to the control group.

The CsAds showed strong chemotactic movement toward bile, specifically in a concentration-dependent manner up to 0.5% bile (Fig 2). The CsAds moved a total of 31.7 ± 4.3 mm in 180 min toward 0.5% bile; in the absence of stimulant, the CsAds remained at the starting line. However, as the concentration increased above 0.5%, the migration distance of the CsAds became shorter and shorter until concentrations reached 10%, which resulted in a slight backwards movement. When the bile concentration was higher than 10%, the CsAds curled up, became damaged and motionless, and died quickly at the starting line.

**Fig 3:**
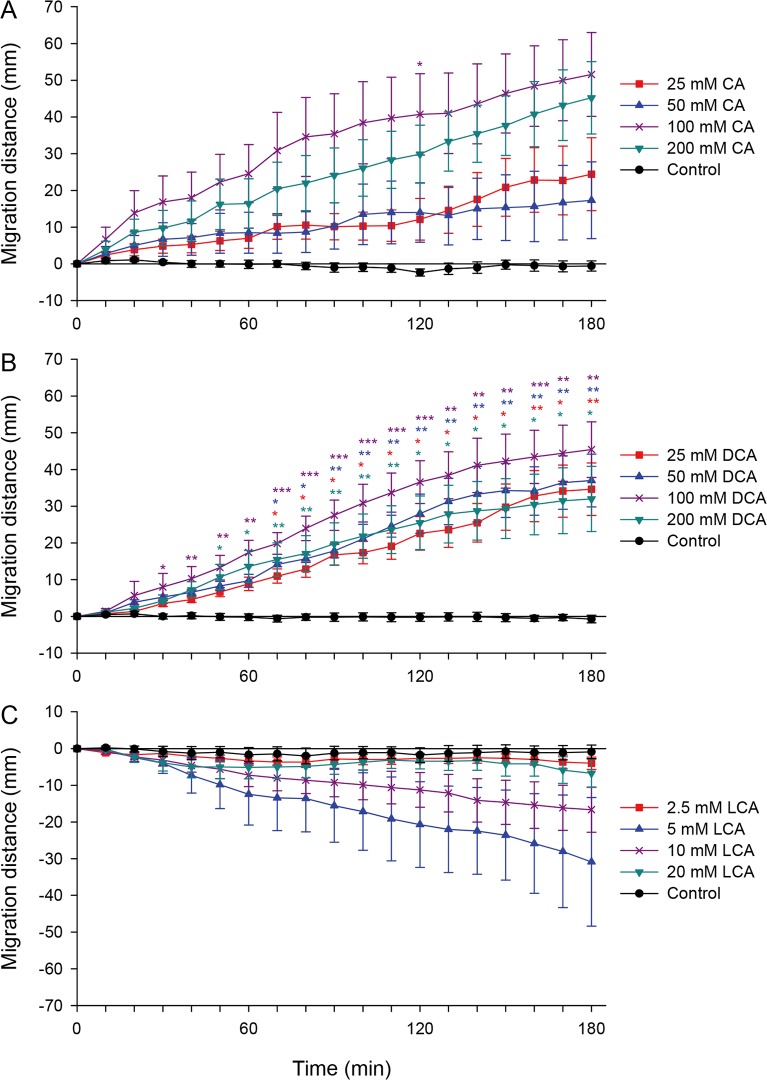
Chemotaxis of CsAds to bile acids. Chemotactic migration distance of CsAds in response to (A) cholic acid (CA), (B) deoxycholic acid (DCA), and (C) lithocholic acid (LCA) was measured every 10 min for 3 h. Asterisks indicate * *p* < 0.05, ** *p* < 0.01, and *** *p* < 0.001 compared to the control group.

The CsAds were attracted with strong chemotaxis to CA and its dehydroxylated metabolite DCA (Fig 3A and 3B). The strongest attraction was induced with 100 mM of both bile acids (S1 Movie). The CsAds moved 51.6 ± 11.4 mm and 45.4 ± 7.6 mm toward 100 mM CA and DCA, respectively, in 180 min. However, the migration distance was shorter toward 200 mM of both bile acids.

In contrast to CA and DCA, LCA acted as a repellent to CsAds (Fig 3C). The CsAds moved in the opposite direction from LCA, and at 5 mM LCA the migration distance peaked at −30.9 ± 17.5 mm in 180 min. There was a vast individual difference in the migration distance. At concentrations higher than 5 mM LCA, however, the CsAds migrated over shorter and shorter distances. At 5 and 10 mM LCA, a third of CsAds turned their heads back from LCA and moved in the opposite direction (S1 Movie), but the remaining two-thirds just moved back. In response to 20 mM LCA, the CsAds were repellent for the first 60 min, but then shrank and remained still.

### Dopaminergic antagonists inhibited chemotaxis of *C*. *sinensis* adults

Previously, we reported that certain dopaminergic antagonists were potent inhibitors of the chemotactic migration of CsNEJs toward CA [13]. Here, their effects were reevaluated on the CsAds.

**Fig 4:**
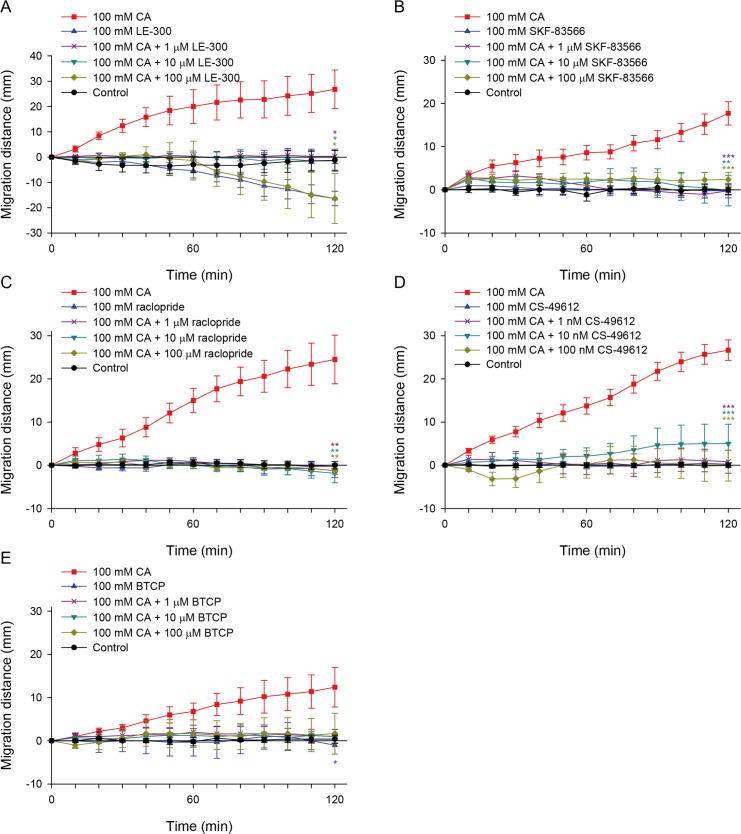
Dopaminergic antagonists inhibit CsAd chemotaxis to cholic acid. One CsAd in each trough was incubated in 1× Locke’s solution containing LE-300 (A), SKF-83566 (B), raclopride (C), CS-49612 (D), or BTCP (E), for 10 min at the starting line, and stimulated with 100 mM CA. The migration distance of the CsAds was measured every 10 min for 2 h. Each chemical, at 100 mM, was also tested as a stimulant to determine its own chemotactic effect (blue triangle up). Asterisks indicate * *p* < 0.05, ** *p* < 0.01 and *** *p* < 0.001 compared to the CA-only control (red square) at 120 min.

A dopamine D_1_ receptor antagonist, LE-300, had its own repellent effect on CsAds. When the CsAds were stimulated with 100 mM LE-300, they moved away from the starting line (Fig 4A). Chemotactic attraction of the CsAds to 100 mM CA was completely negated when they were incubated in 1 and 10 μM LE-300. They did not move in either direction and remained at the starting line. In the presence of 100 μM LE-300, however, flukes moved away from CA just as much as they did when stimulated with LE-300 only.

Another D_1_ receptor antagonist, SKF-83566, and a D_2_/D_3_ receptor antagonist, raclopride, at 1–100 μM, almost completely inhibited the chemotaxis of CsAds toward 100 mM CA (Fig 4B and 4C).

As dopaminergic receptor antagonists inhibited chemotaxis of the CsAds toward CA, chemicals with similar molecular structures were obtained through ChemSpider (http://www.chemspider.com/). Of the twenty chemicals identified, only CS-49612, a derivative of raclopride, was commercially available through Chembo Pharma (http://www.chembopharma.com/). CS-49612 more potently inhibited chemotaxis of the CsAds toward CA at nanomolar concentrations (1–100 nM) (Fig 4D). In addition, CS-49612 agitated the CsAds and led to hyperactivity. Unexpectedly, a dopamine re-uptake inhibitor, benzothiophenylcyclohexylpiperidine (BTCP), also suppressed the chemotaxis of the CsAds at 1–100 μM (Fig 4E).

Unlike LE-300, these four chemicals did not induce any chemotactic responses in the CsAds. The CsAds did not show any notable change in body shape and maintained normal activity in the presence of the dopaminergic antagonists tested, aside from CS-49612. With CS-49612, some flukes curled to round or semi-round shapes at micromolar concentrations; some overreacted and moved quickly forward or backward in a concentration-independent manner.

### Antipsychotic drugs inhibited chemotaxis of *C*. *sinensis* adults

**Fig 5:**
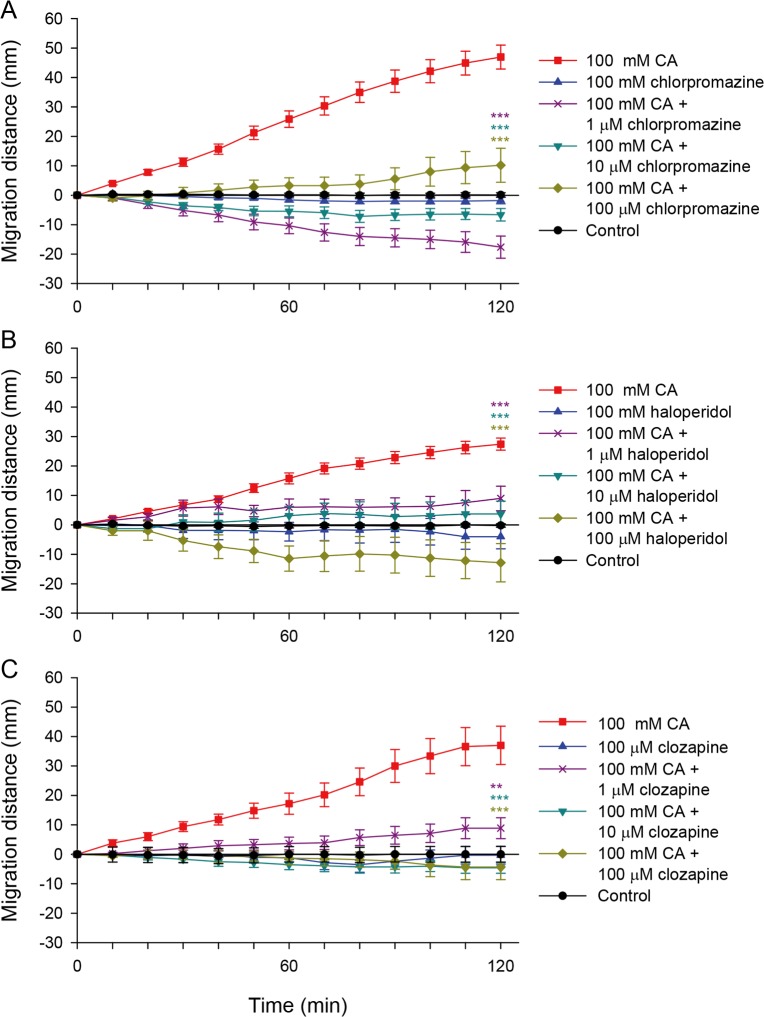
Effects of clinical antipsychotic drugs on chemotaxis of CsAds to cholic acid. The CsAds were stimulated with 100 mM CA solution containing chlorpromazine (A), haloperidol (B), or clozapine (C). The migration distance of the CsAds was measured every 10 min for 2 h. Each drug at 100 mM (except clozapine at 100 μM) was tested for chemo-attractive or chemo-repellent properties (blue triangle up). Asterisks indicate ** *p* < 0.01 and *** *p* < 0.001 compared to the CA-only group (red square) at 120 min.

The dopaminergic receptor antagonists are clinically used for antipsychotic, antiemetic, and gastroprokinetic purposes. Of the antipsychotic drugs, 3 dopaminergic receptor antagonists, namely chlorpromazine, haloperidol, and clozapine, are secreted into bile [16–18]. Their ability to inhibit the chemotaxis of CsAds toward CA was examined. All three drugs significantly suppressed CA-induced chemotaxis of the CsAds at micromolar concentrations *in vitro* (Fig 5).

Chlorpromazine inhibited chemotaxis of the CsAds toward CA (Fig 5A). When stimulated with 100 mM CA plus 1 μM chlorpromazine, half of the CsAds slowly regressed backwards, 40% moved rapidly in the opposite direction, and the rest did not move at all. With 10 μM chlorpromazine, one-third of CsAds moved away rapidly, but the rest did not move. With 100 μM chlorpromazine, however, 40% of the CsAds moved rapidly toward CA, whereas the rest did not move. In general, the CsAd behavior was erratic, sometimes hyperactive and aimless. As the concentration of chlorpromazine in CA solution increased to the millimolar range, some flukes shrank a little or curled up, and showed weak motility. When 100 mM chlorpromazine alone was used for stimulation, the CsAds did not move in either direction, and remained at the starting line.

Haloperidol strongly inhibited chemotaxis of the CsAds toward CA (Fig 5B). When stimulated with 100 mM CA plus 100 μM haloperidol, the CsAds moved rapidly in the opposite direction to CA for the first 1 h and then stopped. With 10 μM haloperidol, the CsAds moved a short distance toward CA for the first 1 h and then stopped. With 1 μM haloperidol, the CsAds moved slowly toward CA for 30 min, and then did not move anymore. Haloperidol at 100 mM alone did not stimulate the CsAds to move. Most of the affected CsAds displayed normal morphology but some curled into S-shapes.

Clozapine strongly inhibited the chemotaxis of the CsAds toward CA (Fig 5C). When stimulated with 100 mM CA and 10 or 100 μM clozapine, about 30% of the CsAds moved a short distance in the opposite direction, but the rest did not move. With 1 μM clozapine, half of the CsAds moved very slowly toward CA, while the other half did not move. Clozapine alone at 100 μM did not stimulate the CsAds to move.

### Worm expulsion and egg reduction

**Fig 6:**
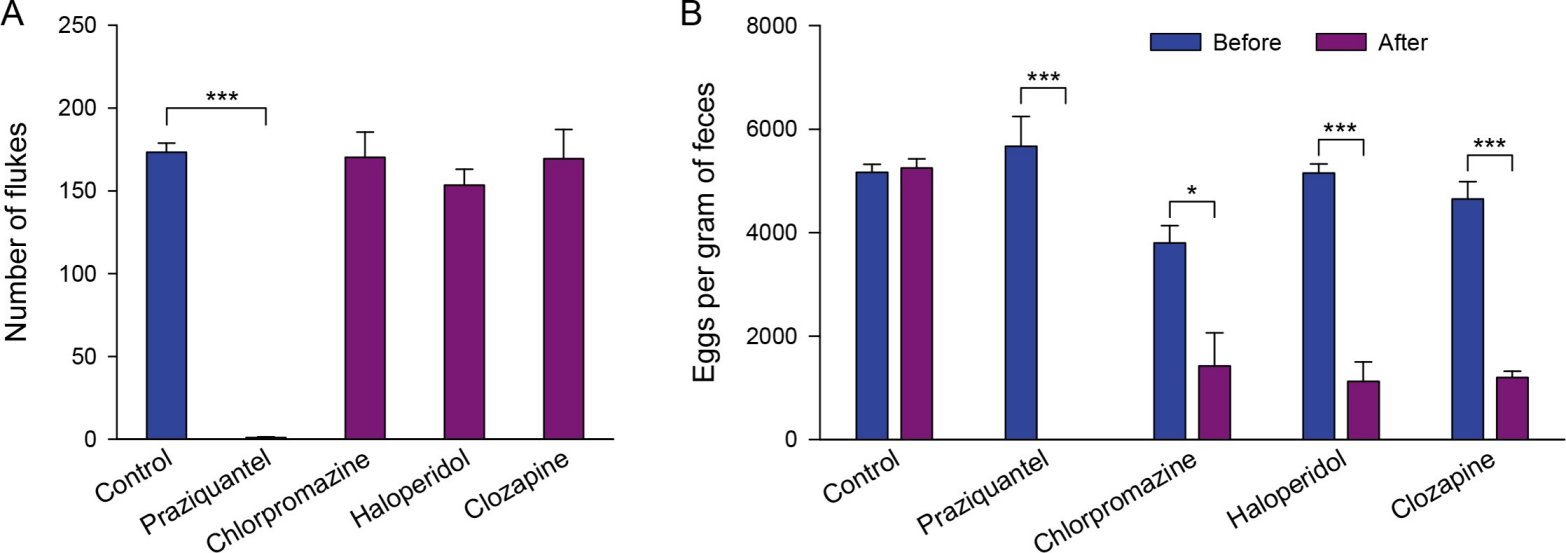
Effects of clinical antipsychotic drugs on worm expulsion and egg reduction. (A) The *C*. *sinensis* adult flukes recovered from the experimental rabbit bile ducts. (B) Eggs per gram of feces before and after drug administration. Asterisks indicate * *p* < 0.05 and *** *p* < 0.001.

Effects of the three clinical antipsychotic drugs on worm expulsion and egg reduction were examined in rabbits infected with *C*. *sinensis* (Fig 6). After 5 days of the last drug administration, rabbits were sacrificed and the CsAds were recovered from the bile ducts. All the three antipsychotic drugs were not able to expel the CsAds from the bile ducts. The worm expulsion rate of haloperidol was 12%, higher than that of chlorpromazine and clozapine, which were both at 2% (Fig 6A). In the positive control group treated with praziquantel, almost all CsAds (99.4%) were expelled. The remaining CsAds in praziquantel group found all emaciated, slender and smaller than the normal, and white in color.

*C*. *sinensis* eggs per gram of feces (EPG) were counted before and after drug administration. In contrast to their negligible expulsion of *C*. *sinensis* adult worms from the bile ducts, the three antipsychotic drugs significantly reduced the number of *C*. *sinensis* eggs present in rabbit feces. The egg reduction rate of haloperidol was 79%, followed by clozapine and chlorpromazine at 74% and 64%, respectively (Fig 6B).

## Discussion

Organisms living in certain hosts or environments possess a trait of chemotaxis to unique stimulants. The CsAds were expected to exhibit bile-chemotaxis since they live inside the bile ducts. Our previous study demonstrated that CsNEJs were chemotactically attracted toward bile and CA but repelled from LCA [12]. Furthermore, the chemotaxis of CsNEJs toward CA was inhibited by dopaminergic antagonists [13]. The current study was carried out to investigate the chemotaxis of CsAds to bile and bile acids, and its dopaminergic regulation.

The migration distance of the CsAds toward bile increased in a concentration-dependent manner, up to 0.5% bile. However, as the concentration of bile increased, the CsAds migrated less and less, and eventually moved away from 10% bile. The chemotactic behavior of CsAds in response to bile was similar to that of CsNEJs [12], but CsAds were less repelled by the higher concentrations of bile. This kind of chemotactic preference to a specific concentration was also observed in the infective third-stage larvae of *Strongyloides ratti*. The *S*. *ratti* larvae preferred 80 mM NaCl and exhibited avoidance to concentrations either below or above 80 mM NaCl [19]. Of the bile acids, CsAds showed the strongest chemotaxis to 100 mM CA and DCA, and less to higher and lower concentrations of these bile acids. Similar to the CsNEJs being repelled by LCA, the CsAds moved rapidly in the opposite direction from 5 mM LCA, but their movement slowed down, and some died when exposed to 20 mM LCA. In contrast to the CsNEJs, the CsAds showed significant chemo-attraction toward DCA *in vitro*. DCA-chemotaxis of the CsAds is unique and differs from the CsNEJs being preferential only to CA [12]. The CsNEJs excyst from the metacercariae, resting and maintaining basal physico-metabolism in a confined environment, and then rush into the bile duct. The CsNEJs may need a chemical cue (such as CA) to migrate quickly to the bile duct by chemotaxis. As the body size of the CsAd increases, male and female reproductive organs are formed, and vast array of physico-metabolic pathways are initiated [20]. As resultant products, the adult flukes excrete a large amount of eggs into the bile duct. It is suggested that CsAds may need stronger chemotactic stimuli to keep the bigger body in the bile duct and to maintain reproductive function. This notion deserves further investigation into the CsAd responses to DCA-induction and stimulation.

Dopaminergic neurons are colocalized with cholinergic neurons in the somatic muscle tissues of the CsAds, and dopaminergic antagonists strongly suppress chemotaxis of the CsNEJs toward bile and CA [13]. In the present study, chemotaxis of the CsAds toward bile and CA was inhibited by dopaminergic antagonists. Dopamine D_1_ receptor antagonists LE-300 and SKF-83566, and D_2/3_ receptor antagonist raclopride, all at 1 μM, almost completely suppressed the chemotaxis of CsAds toward CA. CS-49612, a derivative of raclopride, was even more potent to inhibit chemotaxis at a low nanomolar concentration. A dopamine re-uptake inhibitor, BTCP, also strongly suppressed chemotaxis. This suppression/inhibition was also observed on the chemotaxis of the CsNEJs [13]. In terms of concentration, the chemotactic behavior of CsAds was more susceptible to the suppressive effects of dopaminergic antagonists than that of the CsNEJs.

Praziquantel has been used as a choice of anthelmintic drug for *C*. *sinensis* [21]. However, there are reports that *C*. *sinensis* and schistosomes are less susceptible to praziquantel treatment [22, 23]. Thus, it is important to explore efficacious anti-*C*. *sinensis* drug candidates. Drug repositioning is a powerful technique used to predict new efficacies of existing drugs [24]. There have been several trials to explore alternative anti-trematode drugs, such as artemisinins, mefloquine, and tribendimidine against clonorchiasis, schistosomiasis, and opisthorchiasis, respectively [25–28].

As dopamine antagonists were found to be strong suppressors of chemotactic behavior in both CsNEJs and CsAds, it was postulated that antipsychotic drugs with dopamine-antagonist activity could be effective in preventing *C*. *sinensis* infection. When a drug is secreted into bile, it could more effectively and directly interact with *C*. *sinensis* for the sake of anthelminthic efficacy. Taking this into account, antipsychotic drugs secreted into the bile: chlorpromazine, haloperidol, and clozapine were selected, and their efficacy was examined *in vitro* and *in vivo*. The three antipsychotic drugs suppressed chemotaxis of CsAds to CA as strongly as the chemical dopamine antagonists did. The suppressive effects of haloperidol and clozapine were more or less concentration-dependent. However, chlorpromazine, at a low micromolar concentration, induced the CsAds to move away from CA. The CsAds appeared excited and disoriented, as if chlorpromazine had disturbed their neuro-muscular signaling. In terms of worm expulsion potential, the three antipsychotic drugs were ineffective in dislodging the CsAds from the rabbit bile ducts. It was speculated that either the metabolite forms of the drugs lost the inhibitory property to bind to dopaminergic neuro-receptors, or the amount of the drugs excreted into the bile was insufficient to inhibit the chemotactic behavior of the CsAds in the bile ducts.

The three antipsychotic drugs all significantly reduced the egg production of the CsAds, in terms of EPG, in the infected rabbits. EPG is correlated to worm burden (number of CsAds) in patients with clonorchiasis and used as an index of the infection intensity [15, 29]. The prominent reduction in egg production was believed to be due to the antipsychotic drugs disrupting the reproductive machinery of the CsAds. Although the CsAds still remained in the bile ducts after treatment with the antipsychotic drugs, they were dwarfs and were pale in color, showing a serious impairment in physiology and reproduction [29].

Collectively, we demonstrated that the bile acids CA and DCA were chemo-attractants to CsAds, but LCA was a chemo-repellent. Chemotaxis of CsAds toward CA was suppressed by chemical dopaminergic antagonists. The three antipsychotic drugs with dopamine antagonist-activity, namely chlorpromazine, haloperidol and clozapine, suppressed the chemotactic movement of CsAds. These drugs significantly suppressed egg production of the CsAds in the experimental rabbits. In general, antipsychotic drugs are associated with extrapyramidal side effects, movement disorders, and metabolic disorders in the course of schizophrenic patients treatment [30]. Therefore, the anthelminthic efficacy of dopaminergic antagonists toward *C*. *sinensis* needs to be investigated further.

## Supporting information

S1 Fig Survival rate and activity of *C*. *sinensis* adults. Five CsAd flukes were incubated in 1× Locke’s solution, and their survival and activity were examined under a stereomicroscope for 126 h. The experiment was performed in duplicate.
(TIF)

S2 Fig Bile regurgitation from *C*. *sinensis* adults. Five CsAd flukes were incubated in 1× Locke’s solution for 66 h. The incubation solution was removed and replaced with fresh solution at regular intervals. Bile acid concentration in the solution was determined colorimetrically by measuring absorbance at 546 nm.
(TIF)

S1 Movie Chemotactic responses of CsAds to cholic acid and lithocholic acid. Adult *C*. *sinensis* moved toward cholic acid (CA 100 mM), but away from lithocholic acid (LCA 5 mM). Note that fluke exposed to LCA flipped over and moved away. Fluke that was not exposed to a stimulant remained at the start line for the entire time (-). Video was compressed to 17 s from actual time of 39 min.
(MP4)
