# Supplementary figures and images for: Dopaminergic antagonists inhibit bile chemotaxis of adult Clonorchis sinensis and its egg production
Source: PLoS Negl Trop Dis. 2020 Mar 30;14(3):e0008220. doi: 10.1371/journal.pntd.0008220 (PMC7145267; doi:10.1371/journal.pntd.0008220)

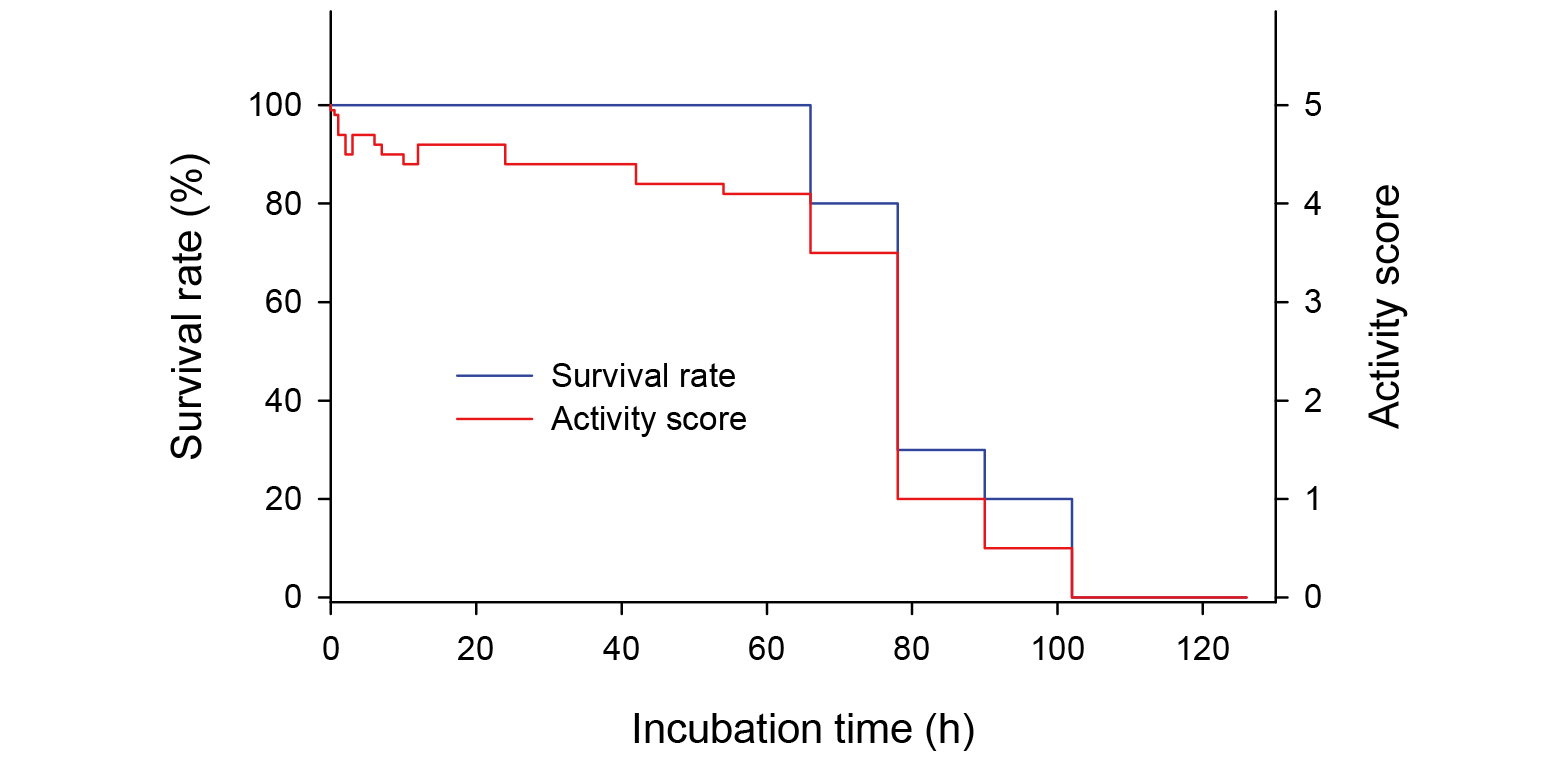

Supplement: S1 Fig — Five CsAd flukes were incubated in 1× Locke’s solution, and their survival and activity were examined under a stereomicroscope for 126 h. The experiment was performed in duplicate. (TIF) [file pntd.0008220.s001.tif]

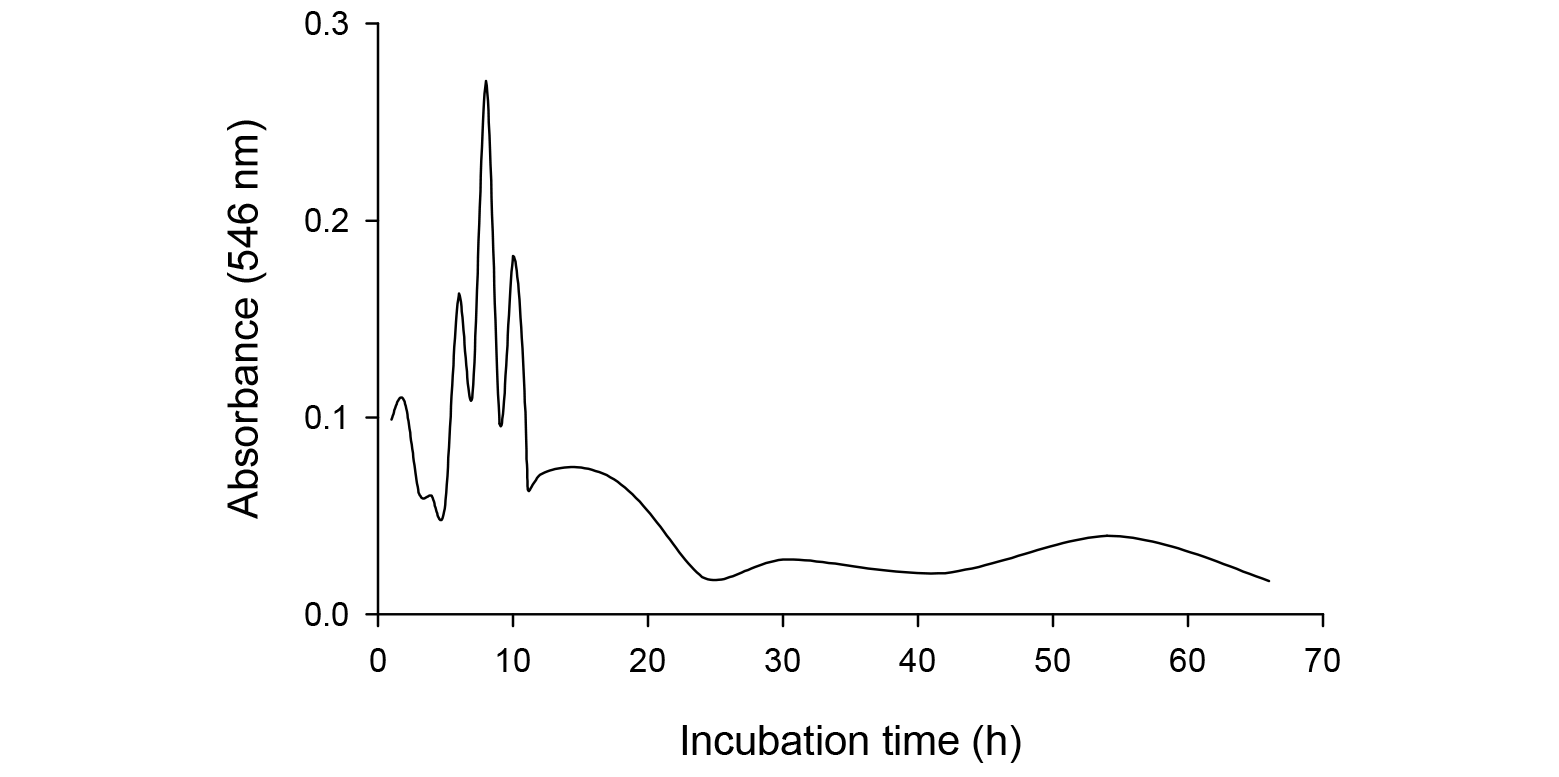

Supplement: S2 Fig — Five CsAd flukes were incubated in 1× Locke’s solution for 66 h. The incubation solution was removed and replaced with fresh solution at regular intervals. Bile acid concentration in the solution was determined colorimetrically by measuring absorbance at 546 nm. (TIF) [file pntd.0008220.s002.tif]
